# Varying rotation lengths in northern production forests: Implications for habitats provided by retention and production trees

**DOI:** 10.1007/s13280-017-0909-7

**Published:** 2017-02-24

**Authors:** Adam Felton, Johan Sonesson, Urban Nilsson, Tomas Lämås, Tomas Lundmark, Annika Nordin, Thomas Ranius, Jean-Michel Roberge

**Affiliations:** 1 Southern Swedish Forest Research Centre, SLU, Box 49, Rörsjöv 1, 230 53 Alnarp, Sweden; 2 Skogforsk, Science Park, 75183 Uppsala, Sweden; 3 Department of Forest Resource Management, Swedish University of Agricultural Sciences, 901 83 Umeå, Sweden; 4 SLU, Skogsmarksgränd, 901 83 Umeå, Sweden; 5 Department of Ecology, SLU, Box 7044, 750 07 Uppsala, Sweden; 6 National Inventory of Landscapes in Sweden (NILS), Department of Forest Resource Management, Swedish University of Agricultural Sciences (SLU), 901 83 Umeå, Sweden; 7 Department of Wildlife, Fish and Environmental Studies, SLU, 901 83 Umeå, Sweden

**Keywords:** Dead wood, Large trees, Rotation length, Thinning, Tree retention

## Abstract

Because of the limited spatial extent and comprehensiveness of protected areas, an increasing emphasis is being placed on conserving habitats which promote biodiversity within production forest. For this reason, alternative silvicultural programs need to be evaluated with respect to their implications for forest biodiversity, especially if these programs are likely to be adopted. Here we simulated the effect of varied rotation length and associated thinning regimes on habitat availability in Scots pine and Norway spruce production forests, with high and low productivity. Shorter rotation lengths reduced the contribution made by production trees (trees grown for industrial use) to the availability of key habitat features, while concurrently increasing the contribution from retention trees. The contribution of production trees to habitat features was larger for high productivity sites, than for low productivity sites. We conclude that shortened rotation lengths result in losses of the availability of habitat features that are key for biodiversity conservation and that increased retention practices may only partially compensate for this. Ensuring that conservation efforts better reflect the inherent variation in stand rotation lengths would help improve the maintenance of key forest habitats in production forests.

## Introduction

Biodiversity refers to the encompassing variability among all living organisms, including diversity within species, between species, and of ecosystems (CBD 1992). Recognition of the role that biodiversity plays in ecosystem function (Thompson et al. 2011; Cardinale et al. 2012), and concerns regarding global losses of forest biodiversity (CBD 2010), is motivating efforts to increase the availability of habitats promoting biodiversity within highly modified production forests (Bengtsson et al. 2000; Lindenmayer et al. 2006). These efforts include the use of retention practices, whereby living and dead trees are retained at final harvest (Gustafsson and Perhans 2010; Lindenmayer et al. 2012a; Johansson et al. 2013). The specific aim of such practices is to increase the availability of key habitats often lacking in intensively managed production stands, including old large trees (Lindenmayer et al. 2012b, 2014), dead wood (Stokland et al. 2012), and tree species of especially high value to flora and fauna (Berg et al. 1994). Whereas retention practices are effective at increasing the provision of such habitats (Fedrowitz et al. 2014), it remains unclear how these efforts may interact with silvicultural programs which alter habitat availability in the harvested portion of the stand.

In even-aged forest management systems, a key component of silvicultural programs is the rotation length, i.e., the time elapsed between two final felling events. The rotation length is a substantial determinant of habitat availability in stands, because it affects the size and age of the trees, the size and amount of dead wood, stand micro-climate and understorey conditions, and the time period available for colonization and establishment by forest-associated species (Roberge et al. 2016). The most widely used method to determine a stand’s rotation length is to optimize the so-called “land expectation value” (LEV), which is the discounted value of the forest following a sequence of identical rotations (Faustmann 1849). This in turn is strongly influenced by wood volume growth, which in even-aged stands follows a pattern whereby the current annual increment (CAI) increases after stand establishment, peaks when maximum leaf area is reached, and then slowly declines (Assmann 1970). Combining these general growth patterns with the Faustmann formula has the added implication that the economic interest rate is a key determinant of rotation length, whereby higher interest rates promote shorter rotations and vice versa.

Decisions about rotation lengths may also be influenced by the desire to increase biodiversity, or reduce the risks posed by storm damages, or pest and pathogen outbreaks (Roberge et al. 2016). The use of genetically improved planting material is also reducing rotation lengths relative to those applied in the past, as such material results in increased tree growth rates (Simonsen et al. 2010). Adaptive responses to climate change can likewise reduce rotation lengths (Felton et al. 2016), due to improved growing conditions (Bergh et al. 2010), or alternatively by strengthening the motivation to harvest earlier due to increased risks of disturbance events (Valinger and Fridman 2011; Bergh et al. 2012). In contrast, management designed to help mitigate climate change may lead to increased rotation lengths, in order to enhance the carbon storage capacity of production forests (Liski et al. 2001; Kaipainen et al. 2004; Zanchi et al. 2014). Consideration of recreational values may also result in increased rotation lengths, if public preferences for larger trees, or dislike for clearcuts, influence harvesting decisions (Gundersen and Frivold 2008). In addition, it is important to note that changes to rotation lengths cannot be considered in isolation from thinning programs, with respect to their either economic or biodiversity implications, since each silvicultural decision is usually dependent on the other. In general, the number of pre-commercial and commercial thinnings will increase with longer rotation periods (Roberge et al. 2016).

Here we examine the impact of different rotation lengths and associated thinning programs on the availability of structures of demonstrated importance to forest biodiversity. We use even-aged boreal forests in Sweden managed by clear-felling and examine the implications of rotation lengths ranging from approximately 50 to almost 200 years for three key habitat indicators (i.e., tree size, availability of dead wood, and tree species composition), often found to increase stand-level species richness or abundance of both common and threatened forest taxa. Our focus on habitat indicators is consistent with the ‘coarse filter’ approach to the conservation of forest biodiversity (sensu Hunter et al. 1988), whereby those production forest stands which better match natural processes of growth and decay are expected to provide habitat features which ‘capture’ a higher proportion of native forest taxa, than production stands that lack these structures and processes (see also ‘natural disturbance emulation’; Angelstam 1998; Kuuluvainen 2002; Lindenmayer and Franklin 2002). In Fennoscandia and other forested regions where protected area networks are insufficient on their own to secure viable populations of native forest species, improving habitat availability in production forests can make an important contribution to the conservation of forest biodiversity (Kuuluvainen 2009; Gustafsson and Perhans 2010). To assess the implications of different rotation lengths, we model the development of stands of Norway spruce (*Picea abies*) and Scots pine (*Pinus sylvestris*) on high- and low-productive sites. As a tool to model different rotation lengths, we vary the interest rate. This approach enables the realistic optimization of different rotation lengths and associated thinning programs. The primary objectives of the study include (1) assessing how indicators of habitat quality vary in relation to rotation lengths, site productivity, and the dominant tree species and (2) highlighting the interaction between conservation interventions and rotation lengths and their joint importance for meeting biodiversity goals in production forest landscapes.

## Materials and methods

### Modeling procedures

To examine the effect of rotation lengths and associated thinning regimes on the availability of habitats promoting biodiversity, we modeled different silvicultural programs (thinning program and time for final felling) obtained by optimizing the LEV according to Faustmann (1849) for interest rates between 0 and 5% with 1% increments. We applied the Heureka model, which is the most developed and used forestry planning and optimization tool in Sweden (Wikström et al. 2011) and calculated economically optimal management protocols for four production forest types in Sweden. We modeled two even-aged stands of Norway spruce: one on a high-productive site [Site index (SI) = 30 m] and another on a low-productive site (SI 18 m). We also modeled two even-aged stands of Scots pine, on high-productive (SI 24 m) and low-productive (SI = 16 m) sites. Site index is determined by top height at the age of 100 years, where top height is the average height of the 100 trees ha^−1^ with the largest diameter at breast height (dbh) (Elfving and Kiviste 1997). Site index has been shown to correlate well with potential production (Ekö et al. 2008). These site indices were chosen in order to represent high- and low-productive site conditions for areas where Scots pine and Norway spruce dominate in Sweden. Although these stands involve the targeted production of single tree species, they are not pure monocultures: natural regeneration and the targeted retention of other tree species result in more diverse tree species composition.

The simulations were done in two steps. First, we used the standard procedure for treatment program optimization in the Heureka software (Wikström et al. 2011) to determine rotation lengths and associated thinning regimes for the chosen interest rates. The procedure computes a large set of management alternatives for each stand with respect to the timing of final felling and the number and timing of thinnings. In all cases, ‘thinning from below’ was simulated and thinning intensity involved the removal of between 25 and 40% of basal area. In the simulations, broadleaved trees had a higher probability of removal during thinning operations, but the broadleaved proportion was nevertheless retained above 10% during the entire rotation. The different management alternatives were ranked according to the LEV and the alternative with the highest LEV was chosen for further analysis. Thereafter, the chosen rotations with associated thinning regimes (silvicultural programs) were simulated as described below.

Growth of stands and individual trees was simulated using empirical growth and yield functions (Fahlvik et al. 2014). For stand growth, stand basal area and individual tree diameter are simulated for five-year periods. At the end of each five-year period, individual tree diameters are adjusted to sum up to the stand basal area given by the stand-level function. At the stand level, the model simulates total five-year basal area growth (m^2^ ha^−1^) for all tree species. At the single-tree level, separate models provide five-year diameter growth rates for Scots pine, Norway spruce, birch (*Betula* spp.), and aspen (*Populus tremula*). Both models are empirical and generate growth based on variables describing the site (e.g., latitude, temperature sum, and site index), the stand (e.g., age, basal area, and time since thinning), and individual tree functions (e.g., diameter and age). Validation of both the stand and individual tree growth models were done by Fahlvik et al. (2011), who demonstrated that the outcomes from the model fit well with the observed tree growth. Potential height growth is modeled for individual trees with height growth functions for top-height trees (Elfving and Kiviste 1997). Thereafter, height growth is reduced with a competition modifier depending on the basal area of trees larger than the subject tree. Estimated individual tree diameter and height are used to calculate individual tree stem volume according to volume functions developed by Brandel (1990). Thereafter, individual tree stem volumes are summed to achieve per hectare values. Hereafter, we use the term ‘production trees’ to refer to the trees grown for industrial use, and ‘retention trees’ for the trees retained for biodiversity conservation at clearfelling.

Mortality due to damaging agents such as storm and insect attacks, and self-thinning due to high density, was simulated for all trees. For the retention trees, mortality rates were assumed to be 12% for the first five years after clearcutting and 6% during the subsequent five-year period. Ten years after clearcutting, the mortality model used for production trees was applied to retention trees (Fridman and Ståhl 2001). Increased mortality a few years after clearfelling has been observed in several empirical studies (Hautala and Vanha-Majamaa 2006; Jönsson et al. 2007; Rosenvald et al. 2008; Heikkala et al. 2014). The assumed levels are in line with a study of retention trees in central Sweden, showing a cumulative mortality rate of 18.5% in the 20 years following clearcutting (Hallinger et al. 2016). As Heureka cannot make distinctions regarding the spatial arrangement of retention trees, the chosen values represent a compromise between the mortality rates associated with single-tree versus group retention practices.

To obtain representative amounts of retention trees, three generations of stand simulations (i.e., three rotations) were run, with the third generation used for the analysis. In the first generation, all simulations were done with treatment programs optimized for the same interest rate (IR) of 2%. We therefore created a common starting point for coming generations, with retention trees representative of today’s current practice. Thereafter, a second generation was simulated using the target IR, with this IR repeated for the simulation of the subsequent (third) generation, which then was analyzed. In this third generation, we have a stand that also contains the surviving retention trees from the two previous generations. Using this generation, we analyze the long-term effects on such structures due to our different rotation lengths with associated thinning regimes. In accordance with the widely implemented forest certification standards (Johansson et al. 2013), 10 retention trees per hectare were excluded from harvest at the end of the first generation. At the end of subsequent generations, additional retention trees were excluded from harvest to compensate for mortality among retention trees during the previous rotation. Therefore, each generation started with 10 retention trees. Only aspen, birch, and pine trees were selected as retention trees, with aspen and birch above 20 cm being prioritized (in that order) over pine. According to our selection criteria, if there were fewer than 10 aspens or birches with a dbh over 20 cm, the largest pines were added to achieve 10 retained trees per hectare. Such a prioritization of large broadleaved trees in the selection of individual retention trees is consistent with current practice in Fennoscandia (see also Roberge et al. 2015).

In order to simulate standing and lying dead wood availability (stumps not included), tree mortality was modeled using a two-step approach. In the first step, average mortality for each stand was estimated, and thereafter mortality was distributed to individual trees. Average mortality was modeled with a logistic function where basal area of larger trees, site index, vegetation type, and thinning history are used as explanatory variables (Elfving 2010). Probability of mortality of individual trees was modeled with the tree species-specific logistic functions developed by Fridman and Ståhl (2001). Decomposition of dead wood was modeled using a negative exponential decay rate according to Harmon et al. (2000). Decay was calculated with the function *e* ^−*rT*^ where “*T*” is the time and “*r*” is a tree species-specific constant for decay in biomass and volume. The value of r for decomposition of volume was 0.013 for Norway spruce and broadleaves and 0.010 for Scots pine. As a response variable, we used only hard dead wood (i.e., little decay), which was determined on the basis of density (Sandström et al. 2007). At final felling, 56% of the dead wood was assumed to be destroyed during the harvest operation (Hautala et al. 2004).

For the economic analysis, the income provided by individual trees was calculated according to a price list provided by the Swedish forest-owner association. The price list distinguishes pine, spruce, and birch timber. Sawlog prices increase with diameter, and sawlog prices are higher than pulpwood prices. Logging costs are calculated according to the productivity of the forestry operation and the average per hour cost for harvesters and forwarders (Brunberg 2012a, b). The cost for regeneration was set at 600€ ha^−1^, and pre-commercial thinning was set at 250€ ha^−1^.

### Biodiversity indicators

We used large trees, broadleaf trees, and dead wood as biodiversity indicators since they are key structures for forest biodiversity in Sweden’s production forests (Gustafsson and Perhans 2010; Johansson et al. 2013). Large trees play crucial roles in forest ecosystems due to the habitat they provide, due to their provision of well-developed crowns, complex bark features, hollows, and sap flows (Lindenmayer et al. 2012b; Siitonen and Ranius 2015). Large broadleaved deciduous trees are particularly important in this regard, as these trees provide specific habitat features which were previously more common in many regions of northern Europe, including Sweden (Lindbladh et al. 2014). Their reduced abundance over recent centuries is associated with widespread population declines and increased extinction risk for many forest species (Berg et al. 1994; Fridman 2000; Bernes 2011). Recently conducted quantitative systematic reviews indicate that the biodiversity benefits of retention trees in production forests generally increase with the number of trees retained, though current data are insufficient to identify widely applicable minimum threshold values for how many trees should be retained (Fedrowitz et al. 2014). Nevertheless, current retention practices required even in certified stands in Sweden (FSC; 10 trees ha^−1^) are often considered insufficient for many forest-associated species (Rosenvald and Lohmus 2008; Johansson et al. 2013).

The presence of old and large trees also allows for the creation of larger diameter dead wood and a faster accumulation of dead wood due to higher mortality (Jonsson et al. 2006). Dead wood is a critical resource for a large number of saproxylic species in boreal forests, which represent approximately 20–25% of all forest species in Fennoscandia (Siitonen 2001; Stokland 2003). This includes many threatened species (Berg et al. 1994; Tikkanen et al. 2006), with large-diameter dead wood hosting an even higher number of red-listed species (Jonsson et al. 2006). The current abundance of dead wood in Swedish production forests (8.1 m^3^ ha^−1^) is only a small fraction of the levels found in old growth forests (Gärdenfors 2010). There is a wide variation among saproxylic species in which type of dead wood they use, how much dead wood they require, and which spatial and temporal scales are relevant, and therefore it is misleading to summarize their requirements for long-term persistence in simple figures of cubic meters per hectare (Ranius and Fahrig 2006). Nevertheless, in various studies of boreal forest saproxylic organisms thresholds have been identified to be between 10 and 80 m^3^ ha^−1^, which is much higher than the amounts found in production forests (Müller and Bütler 2010). For these reasons, efforts are made to increase the quantities of dead wood in production forests (Fridman and Walheim 2000; Schroeder et al. 2011).

To aid comparison with previous studies and acknowledge that species vary in their habitat requirements, we report two minimum diameter size thresholds for both dead wood and living trees. Dead wood is reported as the volume (m^3^ ha^−1^) of hard dead wood above 10 and 30 cm in diameter. The density (trees ha^−1^) of large living trees is reported above 30 and 40 cm in dbh. The standing volume (m^3^ ha^−1^) of broadleaved trees, and for trees in general, is also reported. All three indicators were calculated as an average for the third simulated rotation.

## Results

For all stand types, the longest rotations (0% IR) were approximately twice as long as the shortest (5% IR) (Table 1). All silvicultural programs included thinnings except for the two shortest rotations on the low-productivity Norway spruce site. The number of thinnings increased with increasing rotation length except for the low-productivity Scots pine site, which only had one thinning for the longest rotation (191 years). For the high-productivity sites of both tree species, the silvicultural programs became exactly the same for 3 and 4% IRs (Table 1).

**Table 1:** Stand management programs (number and timing of thinnings and length of rotation, harvested volumes (m^3^ ha^−1^), and mean annual increment (MAI; m^3^ ha^−1^ year^−1^) for Norway spruce (NS) and Scots pine (SP) stands on sites with low (NS low, SP low) and high (NS high, SP high) site fertilities. Stand management was optimized according to different interest rates (IRs) ranging from 0 to 5%

Stand	IR	Stand age at intervention (years)				Harvested volume (m^3^ ha^−1^)		MAI (m^3^ ha^−1^ year^−1^)
		Th1	Th2	Th3	FF	Thinning	Final felling	
NS high	5	46			56	111	363	8.5
	4	46	56		66	197	396	9
	3	46	56		66	197	396	9
	2	51	61		76	230	479	9.3
	1	61	71		86	292	517	9.4
	0	61	71	86	106	418	538	9
NS low	5				81	0	185	2.3
	4				86	0	206	2.4
	3	86			101	69	198	2.6
	2	96			116	83	243	2.8
	1	96			131	84	292	2.9
	0	121			176	115	367	2.7
SP high	5	56			66	101	276	5.7
	4	56			71	101	313	5.8
	3	56			71	101	313	5.8
	2	56	76		91	185	357	6
	1	61	81		106	201	418	5.8
	0	76	96		136	239	482	5.3
SP low	5	76			91	58	169	2.5
	4	81			96	64	180	2.5
	3	81			101	64	197	2.6
	2	81	111		126	118	217	2.7
	1	81	111		136	118	242	2.7
	0	131			191	108	322	2.3

**Fig. 1:**
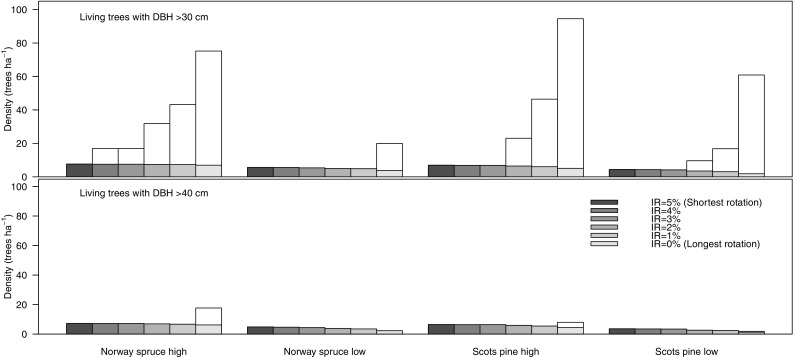
Mean density (trees ha^−1^) of trees with a diameter at breast height above 30 cm (*top*) and 40 cm (*bottom*). The *total bars* represent all trees larger than the diameter limit (including both production trees and retention trees), and the *lower dark part of the bars* represents the contribution of retention trees. The density of large trees was calculated as an average for simulations with an even age distribution of stands of Norway spruce (NS) or Scots pine (SP) in two site productivity classes (high and low) with optimal management according to interest rates (IRs) varying between 0 and 5%. The different IRs were used in order to achieve a gradient in rotation length from the shortest rotation for IR = 5% to the longest for IR = 0% (see Table 1 for details)

The average abundance of production trees with dbh > 30 cm increased with longer rotations in both Scots pine stands and the high-productivity Norway spruce stand (cf. white part of the bars in Fig. 1). For the low-productivity Norway spruce stand, it was only for the longest rotation (176 years) that trees in the production part of the stand reached a diameter >30 cm. Except for the longest rotations in high-productivity stands of both tree species (106 years for spruce and 136 years for pine), trees above 40 cm were only found as retention trees (Fig. 1). The average number of large retention trees decreased with increased rotation lengths for all stands. Even though the absolute decrease in the density of large retention trees with longer rotations was modest, the density was more than halved when comparing short and long rotations in the low-productivity stands (cf. shaded part of the bars in Fig. 1).

**Fig. 2:**
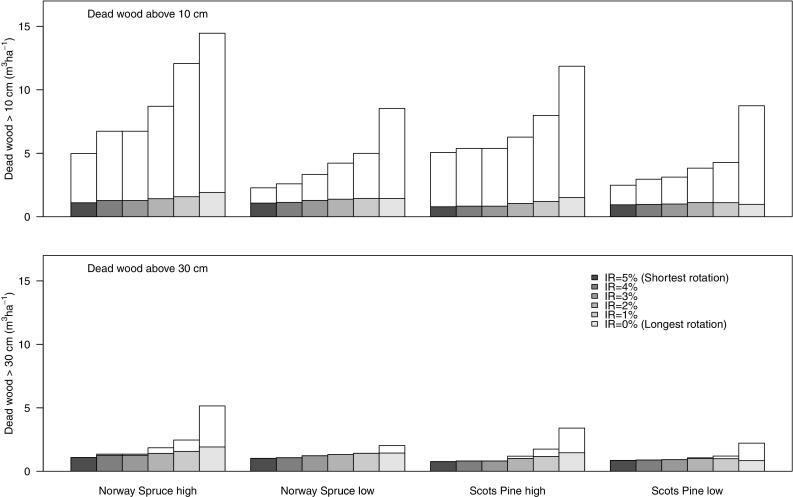
Mean amount of dead wood (m^3^ ha^−1^) in the form of dead trees above 10 cm (*top*) and 30 cm (*bottom*) in diameter. The *total bars* represent all dead wood (including the contributions of both production trees and retention trees), and the *lower dark part of the bars* represents dead wood originating from retention trees. Dead wood was calculated as an average for simulations with even age distribution of stands of Norway spruce (NS) or Scots pine (SP) in two site productivity classes (high and low) with optimal management according interest rates (IRs) varying between 0 and 5%. The different IRs were used in order to achieve a gradient in rotation length from the shortest rotation for IR = 5% to the longest for IR = 0% (see Table 1 for details)

The average amount of dead wood >10 cm in diameter increased with longer rotations (Fig. 2). For short rotations, dead wood from retention trees made up a larger proportion of total dead wood than for long rotations, and this effect was more pronounced for the low- than for the high-productivity sites. Under short rotations, dead wood >30 cm in diameter almost entirely originated from retention trees, whereas under long rotations production trees also contributed to this pool of dead wood (Fig. 2).

**Fig. 3:**
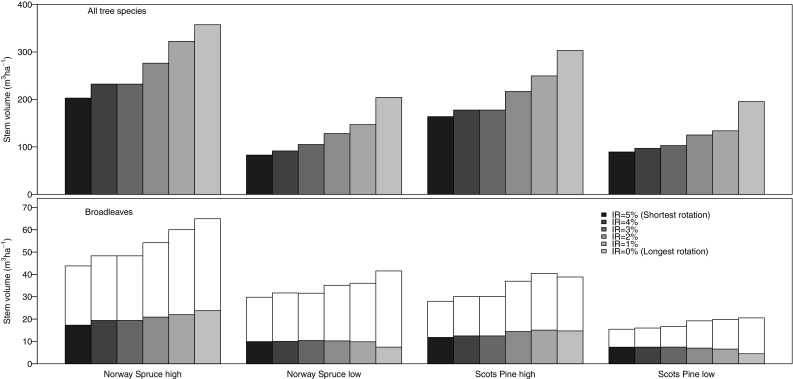
Mean standing volume (m^3^ ha^−1^) of all trees (*top*) and broadleaved trees (*bottom*). For broadleaves, the *total bars* represent all broadleaved trees (including both production trees and retention trees), and the *lower dark parts of the bars* represent retention trees. Standing volume was calculated as an average for simulations with even age distribution of stands of Norway spruce (NS) or Scots pine (SP) in two site fertility classes (high and low) with optimal management according interest rates (IRs) varying between 0 and 5%. The different IRs were used in order to achieve a gradient in rotation length from the shortest rotation for IR = 5% to the longest for IR = 0% (see Table 1 for details)

Average standing volume of broadleaves increased with longer rotations and with increasing productivity, as was the case for trees of all species pooled (Fig. 3). However, the relative contribution of broadleaves to total stand volume increased with shorter rotations. For example, on the low-productivity pine site, the proportion of broadleaves was 11 and 17% for the longest and shortest rotations, respectively. A larger proportion of broadleaves was made up by retention trees in the Scots pine stands compared to the Norway spruce stands (Fig. 3).

## Discussion

Longer rotations were associated with more large trees, dead wood, and broadleaved trees than shorter rotations. This suggests that shortening rotations may be detrimental to habitat availability for a range of species specialized on these structures. Specifically, the shortening of rotations generally reduced the absolute contribution made by production trees to the availability of key habitat features, while concurrently increasing the relative contribution from retention trees. The shortening of rotation lengths eventually reached a point where it prevented production trees from becoming large and thereby providing large coarse woody debris (>30 cm dbh). Once this threshold was crossed, trees and dead wood above 30 cm dbh were only provided by retention trees. The threshold rotation length at which this occurred depended on the dominant tree species and site productivity considered, and ranged from as short as 56 years to more than 130 years. Notably, changes to rotation length generally had a stronger impact on the availability of key habitat features on the most productive sites. On these sites, production trees usually made a more substantial contribution to the abundance of key habitat features than retention trees for long rotation periods. These benefits were lost as rotation periods were shortened.

Despite the same number of retention trees being set aside at final felling, the average density of retention trees varied with rotation length. This is because many retention trees die due to natural mortality (Rosenvald et al. 2008). Whereas these deaths reduce the availability of habitats provided by large living trees, it also increases the availability of dead wood (Vanha-Majamaa and Jalonen 2001; Koskela et al. 2007). In an even-aged forest stand, the retention trees lost during the rotation are not replaced until final harvest. This accounts for the potentially counterintuitive result that the number of large-diameter retention trees (>40 cm dbh) actually increases with shorter rotation lengths, as does the volume of retained broadleaved trees on low-productivity sites. The net result is that shortened rotation lengths increase the frequency at which lost retention trees are replaced. As such, retention practices can reduce the impact of shortening rotation lengths on the availability of some types of habitat.

Retention trees were found to provide certain features, such as trees above 40 cm dbh and coarse woody debris above 30 cm dbh, that production trees provided to only a limited extent. Thus, retention practices will likely favor species dependent on the presence of old large trees and their associated structures, such as tree hollows, crenulated bark, and large branches (Siitonen 2012), as well as species reliant on the larger categories of coarse woody debris (Hyvärinen et al. 2006; Siitonen 2012). The Forest Stewardship Council (FSC) certification in Sweden currently requires a minimum of 10 individual trees be retained per hectare (Johansson et al. 2013). Increasing the number of retention trees in a stand could potentially compensate for the loss of large trees or coarse woody debris caused by shortening rotation lengths, at least for some of the stand types and rotations considered. Although there is no widely applicable minimum threshold for how many trees need to retained, the more general pattern holds that increasing the number of retention trees is often associated with increased biodiversity benefits (Gustafsson et al. 2012; Fedrowitz et al. 2014). Questions can then be raised with respect to the likely production and economic costs associated with increasing retention practices. In this regard, studies of Scots pine-dominated stands in Sweden indicate that retaining 10 trees ha^−1^ reduces wood production by approximately 3% (Jakobsson and Elfving 2004; Elfving and Jakobsson 2006), and that the use of retention practices can be a more cost-efficient way to increase the amount of dead wood in a stand than prolonging the rotation period (Jonsson et al. 2006).

However, since individual retention trees do not provide environments comparable to mature forest conditions (Gustafsson et al. 2010, 2012; Lindenmayer et al. 2012a; Fedrowitz et al. 2014), they cannot fully compensate for shortened rotation lengths, nor can retention trees compensate for the reduced window of opportunity provided to species needing to establish in mature forest stands. Those species which depend on habitats not adequately provided by retention practices will therefore require additional conservation measures (e.g., increased protected areas) to fully compensate for shortened rotation times. In this study, we focused on the implications for production trees and those retention practices directly affected by altered rotation lengths, while assuming that other important aspects of the production forest matrix, such as set asides and buffer zones, remain relatively static. Notably, these additional conservation prescriptions will complement the pool of old trees found within a production stand and may, together with retention trees, help buffer against a decrease in available habitats for biodiversity due to decreased rotation length.

There are several potential reasons for why the rotation lengths used in Sweden’s production forests may be changed (Roberge et al. 2016). In this study, we simulated changes to rotation lengths as if these adjustments were planned for at the beginning of the rotation, using varying interest rates as a catalyst, and assuming that management decisions were guided by the desire to optimize LEV. These circumstances and motivations directly influence outcomes because they allow thinning regimes to be matched to the projected rotation length. Other examples of when thinning regimes may also be matched to rotation lengths include a priori adjustments to reduce expected stand losses to foreseeable disturbance events. This may occur, for example, when recommended rotation lengths are shortened relative to common practice, in order to take into consideration projected increases in storm or pest damages associated with anthropogenic climate change (Felton et al. 2016). In contrast, such alignment between thinning regimes and rotation lengths is less likely to occur in production forests for which the intended rotation length is suddenly cut short due to an unexpected disturbance event. Likewise, our results are less likely to be applicable to those production stands in which rotation lengths are being altered due to improved growing conditions, or the use of faster-growing provenances.

## Conclusion

Due to the range of potential catalysts for altering rotation lengths, and current trends in this regard (Felton et al. 2016; Roberge et al. 2016), it is seemingly unavoidable that the rotation lengths of Sweden’s even-aged stands will change over the coming century. By quantifying the potential effects on the availability of important habitat features from changing rotation lengths, our results should help address a previously identified knowledge gap regarding the potential implications of such changes for forest biodiversity (Roberge et al. 2016). In summary, our results indicate that if rotation lengths are increased, and current retention practices are maintained, the additional key habitats provided by production trees will positively contribute to biodiversity values and help compensate for the attrition of retention trees, which takes place at later stages in the rotation. If, however, rotation lengths are shortened over coming decades, as may occur in Sweden (Södra 2012; Fries et al. 2015), a corresponding reduction in the amount of key habitat features can be expected despite the use of retention practices. For this reason, the inherent variation in stand rotation lengths needs to be better reflected in our conservation efforts, to help ensure that the key forest habitats are maintained in production forests.
